# Human Papillomavirus and the Stroma: Bidirectional Crosstalk during the Virus Life Cycle and Carcinogenesis

**DOI:** 10.3390/v9080219

**Published:** 2017-08-09

**Authors:** Megan E. Spurgeon, Paul F. Lambert

**Affiliations:** McArdle Laboratory for Cancer Research, Department of Oncology, University of Wisconsin-Madison, Madison, WI 53705, USA; megan.spurgeon@wisc.edu

**Keywords:** human papillomavirus, stroma, tumor microenvironment, cervical cancer, paracrine signaling, epithelial–stromal interactions

## Abstract

Human papillomaviruses (HPVs) are double-stranded DNA (dsDNA) tumor viruses that are causally associated with human cancers of the anogenital tract, skin, and oral cavity. Despite the availability of prophylactic vaccines, HPVs remain a major global health issue due to inadequate vaccine availability and vaccination coverage. The HPV life cycle is established and completed in the terminally differentiating stratified epithelia, and decades of research using in vitro organotypic raft cultures and in vivo genetically engineered mouse models have contributed to our understanding of the interactions between HPVs and the epithelium. More recently, important and emerging roles for the underlying stroma, or microenvironment, during the HPV life cycle and HPV-induced disease have become clear. This review discusses the current understanding of the bidirectional communication and relationship between HPV-infected epithelia and the surrounding microenvironment. As is the case with other human cancers, evidence suggests that the stroma functions as a significant partner in tumorigenesis and helps facilitate the oncogenic potential of HPVs in the stratified epithelium.

## 1. Introduction

Viruses are the etiological agents of approximately 15% of human cancers worldwide [1]. There are currently seven identified human tumor viruses, and techniques to discover new viruses associated with human malignancies are continuously emerging and evolving [2]. High-risk human papillomaviruses (HPVs), a group of small DNA tumor viruses that infect the stratified squamous epithelia (referred to hereafter as the stratified epithelium or stratified epithelia), are alone responsible for nearly 5% of worldwide cancers [3]. There are more than 150 types of HPV, and around 40 HPV types are considered mucosotropic in that they preferentially infect the mucosal stratified epithelia of the anogenital tract (cervix, vagina, and anus) and the oral cavity [4,5]. HPV infections are the most common sexually transmitted infection in the U.S., and sexually active individuals are usually infected with HPV at least once during their lifetime [6,7].

However, not all HPV infections cause cancer. HPVs are considered low-risk or high-risk based on their oncogenic potential [8]. Low-risk mucosotropic HPVs cause benign genital warts, whereas those HPV types considered high-risk are causally associated with nearly all cases of cervical cancer and other cancers of the lower female reproductive tract and anus, as well as an increasing proportion of oropharyngeal cancers. The high-risk HPV types HPV16 and HPV18 are particularly formidable, causing the majority of HPV-associated cervical, anal, and oral cancers [9,10,11]. The introduction of prophylactic vaccines that prevent infection by those high-risk HPVs most highly linked to cancers (e.g., HPV16 and HPV18) was a major breakthrough in controlling HPV-associated carcinogenesis [12]. Unfortunately, suboptimal vaccine availability and lagging vaccination coverage continue to make HPV-associated cancers a significant global health issue [13]. A more complete understanding of HPV virus–host interactions during infection and carcinogenesis therefore remains key to identifying new preventative and therapeutic options.

More than 90% of HPV infections in the cervix are naturally cleared [14]. Thus, the factors and mechanisms involved in cases that do ultimately progress to cancer remain the focus of intense research. In the absence of clearance, HPV infections can persist for years or even decades, and these persistent high-risk HPV infections are a major risk factor for subsequent cancer development (Reviewed in [15,16]). While the mechanisms that govern viral persistence and progression to cancer are still unclear, both HPV type and viral load appear to be involved [17,18]. Integration of the HPV viral genome into host DNA is a frequent event during persistent HPV infection and occurs at random sites unique to each cancer [19,20], although a preference for chromosomal fragile sites has been reported [21]. Viral genome integration increases and stabilizes transcription of the viral oncogenes E6 and E7 [22,23] and provides a growth advantage to epithelial cells [24]. HPV E6 and E7 are highly multifunctional proteins that contribute to carcinogenesis, primarily through their ability to inactivate the p53 and retinoblastoma protein (pRb) tumor suppressor pathways [25,26]. Despite its demonstrated role in carcinogenesis, persistent HPV infection is not sufficient for human cervical cancer development [27,28], and thus other co-factors likely contribute to this process.

**Figure 1:**
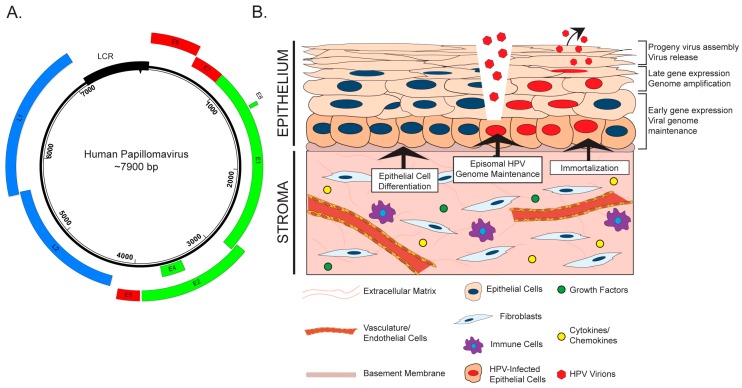
The human papillomavirus (HPV) life cycle and the stroma. (**A**) Map of the HPV genome. Shown is the circular, ~7900 base pair (bp) double-stranded DNA (dsDNA) genome of HPV18. The boxes represent translational open reading frames that encode HPV proteins. Early (E) genes are shown in green and late genes (L) are shown in blue. The long control region (LCR), which regulates transcription and viral DNA replication, is shown by a black box. Three HPV oncogenes, E5, E6, and E7, are shown in red. (**B**) A schematic representation of the stratified epithelium, underlying stroma, and the HPV life cycle is shown. Basal cells, which are adjacent to the basement membrane and underlying stroma, are shown in dark tan. HPV virions infect the basal cells of the stratified epithelium, presumably through a break or wound in the epithelial layer. The virus life cycle proceeds throughout the epithelium and is tied to differentiation, ending with progeny virus production and release from the terminally differentiated cells. Labels indicating the spatiotemporal regulation of key events in the HPV life cycle are shown to the right of the stratified epithelium. The stroma, which is composed of various cell types, vasculature, and connective tissue, has been shown to promote epithelial cell immortalization, maintenance of episomal HPV genomes, and epithelial cell differentiation in support of the HPV life cycle (indicated by black arrows). Please refer to the legend to identify common components of the stroma, and to the text for further detail.

An increasingly recognized co-factor in many types of cancer is the adjacent stroma, or ‘microenvironment’. The stroma is a supportive scaffold upon which epithelial cells reside, and is composed of connective tissue, vasculature, and various cell types (Figure 1). The role of the tumor microenvironment (TME) in carcinogenesis is a relatively nascent, yet growing, area of cancer biology research. Fibroblasts are a major component of the stroma, and tumor-associated or cancer-associated fibroblasts (TAFs or CAFs, respectively) have become a major focus of TME research [29]. Communication between epithelial cells and the TME has been reported to affect processes ranging from tumor initiation and neoplastic progression to metastasis and therapeutic response [30]. HPV-associated cervical carcinogenesis is accompanied by changes in stromal gene expression throughout neoplastic progression, which reflect the likely role of the stroma in supporting angiogenesis and epithelial invasion [31]. However, an in-depth understanding of the interaction between HPV-infected tissue and the surrounding microenvironment has not been achieved. In this review, we explore the current knowledge of the interplay between HPV-positive epithelia and the stroma during viral infection and carcinogenesis. Emerging evidence suggests that these tissue compartments engage in bidirectional crosstalk to facilitate HPV infection and HPV-associated cancers.

## 2. The HPV Life Cycle and the Stroma

HPVs are icosahedral, non-enveloped viruses approximately 50–60 nm in diameter. These viruses contain a double-stranded, circular or episomal DNA genome of approximately 8000 base pairs (Figure 1A). A temporal pattern of viral gene expression gives rise to ‘early’ and ‘late’ genes, which are transcribed using a complex system of promoter usage and splicing patterns [32,33]. HPVs exhibit tropism for the stratified epithelia, and the viral life cycle is intimately tied to the process of cellular differentiation in this tissue (Reviewed in [34]). HPVs infect the poorly differentiated, basal keratinocytes of the stratified epithelium, to which they are believed to gain access through wounds or breaks in the epithelial layer (Figure 1B). Naturally, the basal cells are the only cells actively engaged in cell division within the stratified epithelium. This characteristic of basal keratinocytes helps explain their being targeted by HPV for infection, as both viral genome nuclear entry and genome maintenance require active cell division [35]. Episomal viral genomes are then maintained in basal cells at a low copy number. As infected daughter cells enter the process of terminal differentiation, early viral genes reprogram the parabasal and suprabasal cells to re-enter the cell cycle. A process of viral genome amplification, which is dependent upon host DNA synthesis/repair machinery [36,37], is followed by late gene expression and viral progeny production in and release from the superficial layers of the terminally differentiated stratified epithelium.

The role of the stroma during initial HPV infection and the life cycle is an underexplored area of research. There is evidence that stromal fibroblasts and/or feeder layers, which are comprised of mitotically inactive fibroblasts, affect various stages of the HPV virus life cycle through both direct and indirect mechanisms (Figure 1B). Rheinwald and Green first identified feeder layers as an important component of in vitro keratinocyte culture and propagation [38]. Fibroblasts feeder layers promote epithelial homeostasis and proliferation by providing critical soluble factors, such as growth factors and cytokines, and extracellular matrix components that function in juxtacrine and paracrine manners [39]. There is evidence that feeder layers are necessary for maintenance of the HPV genome, which is replicated and maintained as an extrachromosomal circular plasmid (or episome) in HPV-infected cells. In W12 keratinocytes, which were isolated from an HPV16-infected low-grade cervical lesion, the HPV genome exists in extrachromosomal episomal form. Removal of co-cultured feeder cells from several W12 clonal populations resulted in the gradual loss of HPV episomes and promoted viral genome integration in all clones [40]. Consistent with this finding, unpublished work from our laboratory indicates that high-risk HPV genomes are not maintained in oral keratinocytes in the absence of a feeder layer [41]. Another role for the stroma in the HPV life cycle involves its critical function to facilitate differentiation and stratification of the epithelium through paracrine interactions [42,43], which is essential for completion of the HPV life cycle [44]. To study the complete HPV life cycle, in vitro methods such as three-dimensional organotypic raft culture methods are often used [45,46], which are composed of keratinocytes cultured at the air–liquid interface on top of a dermal equivalent containing fibroblasts [47]. The proper architecture, differentiation, and proliferation of the stratified epithelium in these biomimetic in vitro cultures requires the physical presence of fibroblasts, the reciprocal exchange of diffusible factors, and crosstalk between the two compartments through growth factors and cytokines [42,48,49,50,51,52,53]. Other stromal cell types, such as endothelial cells, also support epithelial differentiation [50,51]. Thus, while the underlying mechanistic understanding is very limited to date, interactions between the stroma and the epithelial compartment clearly contribute to HPV genome maintenance and the differentiation-dependent completion of the HPV life cycle.

## 3. HPV and the Stroma: Bidirectional Paracrine Effects

The stromal microenvironment is a critical, if not wholly essential, participant in several facets of both normal and cancer epithelial tissue biology [29,42]. In the previous section, we discussed the limited knowledge about how stromal cells contribute to HPV infection and the viral life cycle. In the following sections, we will review our current understanding of how HPV-positive epithelia and the stromal microenvironment communicate with and influence one another during HPV-associated disease and cancer development. While, below, we describe the epithelial-to-stromal communication separately from stromal-to-epithelial communication, it should be kept in mind that there likely are complex feedback loops in which signals emanating from one compartment inform on how the other compartment responds back, and that this bidirectional cross talk is likely key to how the viruses manipulate their environment, and in the case of tumor-associated viruses, contribute to carcinogenesis.

### 3.1. Effects of the HPV-Positive Epithelium on the Stromal Microenvironment

**Figure 2:**
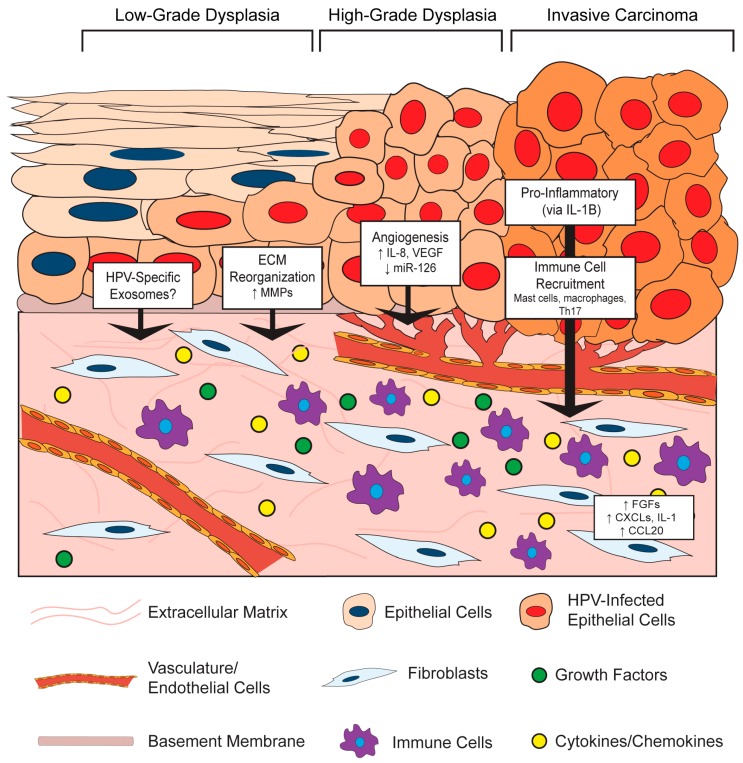
Effects of the HPV-positive epithelium on the stroma. The HPV-infected epithelium undergoes neoplastic progression from low-grade to high-grade dysplasia and eventually invasive carcinoma. Key epithelial-to-stromal communication events between the HPV-positive epithelium and the stroma, as well as the reported paracrine factors involved in these processes, are depicted. Large black arrows indicate events in the HPV-positive epithelium that affect the underlying stroma. Small black arrows represent an increase (up arrow) or decrease (down arrow) in the indicated factor. The location of signaling events within the process of neoplastic progression is not necessarily reflective of their temporal activities. ECM: extracellular matrix; CCL20: C-C Motif Chemokine Ligand 20; CXCLs: C-X-C Motif Chemokine Ligands; FGFs: Fibroblast growth factors; IL-1: Interleukin-1; IL-1B: Interleukin-1B; IL-8: Interleukin-8; miR-126: microRNA-126; MMP: matrix metalloproteinase; Th17: T helper 17 cells; VEGF: vascular endothelial growth factor.

It is increasingly clear that virus-infected cells communicate with the surrounding microenvironment [54]. In the section below, we describe key examples of effects of HPV-positive epithelial cells on stromal architecture, angiogenesis, inflammation, and immune cell recruitment (Figure 2). This epithelial-to-stromal communication likely initiates a process of bidirectional crosstalk and interdependent signaling pathways that persists during HPV-associated disease progression.

#### 3.1.1. HPV Effects on Stromal Architecture

HPV-positive epithelial cells influence the composition and organization of the microenvironment in both in vitro and in vivo models. This paracrine effect of HPV on the underlying stroma may, in part, be due to the ability of E6 and E7 to alter the secretory profile of epithelial cells, which has been shown to regulate the invasive potential of in vitro organotypic raft epithelial cells via signaling between the epithelium and stroma [55]. For instance, HPV16-positive CSCC27 cervical cancer cells promote changes in co-cultured cervical fibroblasts, including the production of a laminin-rich matrix and reduced fibronectin and collagen [56]. Mechanistically, these changes were attributed to reciprocal actions between the HPV-positive epithelial cells and fibroblasts involving matrix metalloproteinases (MMPs): the presence of fibroblasts promoted MMP-7 expression in the epithelial cells, whereas CSCC27 epithelial cells induced MMP-2 expression in the fibroblasts. MMPs are extracellular proteinases with well-known functions in modifying TME architecture, and their enzymatic activities can directly contribute to carcinogenesis by affecting processes such as extracellular matrix reorganization, angiogenesis, and inflammation [57]. Several additional lines of evidence support a role for HPVs in modulating MMP expression. In another in vitro study, HPV18-positive SKG-II cells increased pro-MMP-1 and pro-MMP-3 in co-cultured cervical fibroblasts, presumably through tumor-cell derived soluble factors [58]. Similar findings were reported for HPV16- and HPV18-immortalized cells and HPV-positive cervical cancer cells, where an increase in the membrane-type 1 MMP (MT-1 MMP) as well as MMP-2 was observed, and some evidence suggests that this is a unique function of E7 proteins from high-risk HPV types [59]. Other groups showed that E6 cooperates with E7 to increase MT-1 MMP, MMP-2 and MMP-9 expression in order to enhance epithelial cell stromal invasion and migration [60,61]. Therefore, HPV-positive epithelial cells increase MMP expression, which can contribute to stromal reorganization.

The influence of HPV on the stroma in vivo has been largely investigated through the study of genetically engineered HPV transgenic mice. These in vivo models allow observations within a complete biological system, and are therefore especially critical to understanding the interplay between the epithelium and tumor microenvironment. There are two main HPV transgenic murine models, both of which target HPV genes to the basal cells of the stratified epithelium using the basal keratinocyte-specific keratin 14 (K14) promoter. One model is the *K14-HPV16* model in which the entire HPV16 early region is expressed [62]. In the other model, transgenic mice singly expressing the HPV16 E6 or E7 oncogenes, *K14E6* [63] and *K14E7* [64] mice, which can be crossed to generate bitransgenic *K14E6/E7* mice. In addition to studying cervical cancer, *K14E6*, *K14E7*, and *K14E6/E7* transgenic mice have been used to study HPV-associated cancers at other anatomical sites, including the skin [65], oral cavity [66], and anus [67].

In the skin of *K14HPV16* transgenic mice, the underlying stromal architecture was extensively remodeled during the course of neoplastic progression [68,69]. Architectural changes arose even in premalignant lesions, in the absence of epithelial dysplasia and malignancy, indicating that HPV-positive epithelia can induce reorganization of the microenvironment beginning during the early stages of neoplastic progression. These structural changes included thinning of the basement membrane, apparent degradation and disruption of the collagen fibril network, and additional disintegration of the extracellular matrix [68]. Much of this reorganization was attributed to an infiltration of inflammatory cells, primarily mast cells, and their associated protease activities [69]. Thus, data support a role for HPV in facilitating epithelial-to-stromal signals that result in extracellular matrix reorganization at least in part through HPV-induced MMP expression.

#### 3.1.2. HPV Effects on Angiogenesis in the Stroma

In both the human cervix and the murine cervix of *K14HPV16* mice, angiogenesis and vascular density increases during progression to cancer [70]. Increased vascularity is observed even in early cervical lesions, which implies that HPV infection itself or early consequences of infection promote angiogenesis [71]. HPV-mediated angiogenesis has been directly linked to the functions of the HPV oncoproteins in a variety of in vitro and in vivo studies. In work by Chen et al. [72], conditioned media was collected from human foreskin keratinocytes (HFKs) either transduced with HPV16 E6/E7 or stably transfected with the entire HPV16 genome, or media from the HPV31-positive, cervical intraepithelial neoplasia (CIN) derived cell line, CIN612. Application of conditioned media from these HPV positive cells to endothelial cells in vitro increased their proliferation and migration. This conditioned media was also analyzed in an in vivo Matrigel plug assay, which showed remarkably enhanced vascularization at seven days post-implantation in those plugs composed of HPV-positive media compared to HPV-negative controls. Interestingly, there was a much greater response in vivo, leading the authors to speculate that multiple stromal cell types contribute to this HPV-dependent angiogenic response. Analysis of conditioned media from cells expressing HPV16 E6 identified a significant increase in the pro-angiogenic factors vascular endothelial growth factor (VEGF) compared to that of parental cells [73]. Others observed an increase in VEGF and interleukin (IL)-8 along with reduced expression of angiogenesis inhibitors, thrombospondin-1 and maspin, in human keratinocytes expressing both HPV16 E6 and E7 [72,74] and that expression of both HPV16 E6 and E7 together was necessary to induce angiogenesis [75].

In addition to the secretion of pro-angiogenic factors from HPV-positive epithelial cells that function in a paracrine manner, there is also evidence that HPV-positive cells can stimulate pro-angiogenic gene expression in cells within the adjacent stroma. For instance, CAFs isolated from the stroma of a cervical cancer secreted more VEGF than cervical cancer epithelial cells under both normal and hypoxic conditions [76]. More recently, an intriguing mechanism was reported in which HPV16-positive CaSki cells were found to reduce expression of a micro-RNA (miRNA), miR-126, in endothelial cells [77]. This observation was made using an in vitro tri-culture system composed of CaSki cancer epithelial cells, endothelial cells, and fibroblasts. The reduction of miR-126 in endothelial cells required the presence of both epithelial cells and fibroblasts in the tri-culture, suggesting that a complex network of paracrine interactions is involved in miR-126 regulation. Interestingly, miR-126 was previously identified as a miRNA downregulated in HPV16-positive cervical epithelial cells compared to normal epithelial cells [78], perhaps suggesting that this miRNA is specifically targeted by HPVs. The effect of this miRNA was then investigated using xenografts, which are cells injected subcutaneously into live mice and allowed to generate tumors in vivo. Increased microvasculature density and tube formation was observed in xenografts composed of CaSki cells and CAFs, and this effect was associated with a decrease in miR-126 in host-derived endothelial cells recruited to the tumor. The pro-angiogenic gene for adrenomedullin (*ADM)* and several other pro-angiogenic genes are targets of miR-126 [79]. Adrenomedullin expression was highly increased in the cervical cancer stroma, and its increased expression correlated with miR-126 downregulation in the tri-culture system. Therefore, this fascinating study reveals a cell non-autonomous role of HPV-positive cervical cancer epithelial cells in promoting angiogenesis in the stroma through the regulation of expression of a miRNA in endothelial cells. Exploration of the role of epithelial–stromal crosstalk and the influence of HPV in regulating miRNA expression is only now beginning, but promises to reveal important mechanisms involved in HPV-associated carcinogenesis.

The pro-angiogenic effects of HPV-positive epithelial cells on the stroma have also been observed using in vivo murine models. One such example involves the release of platelet-derived growth factor (PDGF) from the *K14HPV16* cervical epithelium, which leads to expression of fibroblast growth factors 2 and 7 (FGF-2 and FGF-7) in the stroma to promote angiogenesis and tumor cell growth [80]. Fibroblasts isolated from the dermis adjacent to *K14HPV16* murine skin produce several pro-inflammatory cytokines with well-known roles in angiogenesis [81]. When these pro-inflammatory fibroblasts were mixed and co-injected with PDSC5 HPV16-positive skin carcinoma epithelial cells, there was a higher level of vascularization and microvasculature density compared to xenografts with normal fibroblasts. Similar results were observed using an in vivo Matrigel plug assay. Interestingly, this study also found that HPV-positive epithelial cells could ‘educate’ normal fibroblasts in vitro to become pro-inflammatory, and found that this paracrine education is mediated by epithelial-derived interleukin-1β (IL-1B). In a detailed and descriptive study by Coussens et al., important insight is provided into how pro-inflammatory stromal cells function as ‘co-conspirators’ in angiogenesis in the *K14HPV16* epidermis [69]. Beginning early in neoplastic progression, the stroma is characterized by increased capillary density, corroborating other reports that HPV may promote angiogenesis prior to malignant conversion [71]. Throughout neoplastic progression, dermal capillaries become more dilated and enlarged, their density increases, and they become increasingly localized adjacent to the basement membrane. These changes are associated with an increasing pro-inflammatory environment and infiltration of mast cells [69] and macrophages [81]. The mast-cell protease monocyte chemotactic protein-4 (MCP-4), or chymase, was found to be key in promoting an angiogenic switch, as angiogenesis was severely reduced in mast-cell deficient *K14HPV16* mice. Therefore, there are several mechanisms by which the HPV-positive epithelium can instruct changes in fibroblasts, endothelial cells, and other stromal cells that enhance angiogenesis and cancer progression.

#### 3.1.3. HPV Effects on Inflammation and Immune Cell Recruitment in the Stroma

HPVs are notorious for their ability to evade the host immune response during infection, mainly due to the nature of their intraepithelial life cycle that provides ample cover from host detection [82,83]. However, during the course of progression, the microenvironment becomes increasingly associated with an immune or inflammatory infiltrate [71]. Inflammation is involved in several stages of HPV-associated carcinogenesis [84]. While the mechanisms are not completely clear, several studies indicate that HPV-positive epithelial cells direct pro-inflammatory gene expression in fibroblasts or other stromal cell types, which results in immune cell recruitment. For a more comprehensive review of the interaction between HPVs, the immune system, and the stroma, please refer to the review by Woodby et al. [85].

As described in previous sections, a strong pro-inflammatory gene expression signature was measured in fibroblasts isolated from dysplastic skin of *K14HPV16* transgenic mice and was induced by epithelial cell-secreted IL-1B [81]. A prominently represented group of genes within this inflammatory signature included several ELR+ C-X-C family of chemokines (*CXCL1*, *CXCL2*, *CXCL5*), interleukins (*IL-1B*, *IL-6*), the cyclooxygenase-2 gene *COX2*, and several other genes with well-known pro-inflammatory roles. This group of C-X-C motif ligand chemokines (CXCLs), which are all ligands for the CXCR2 receptor, are considered pro-angiogenic and also function as chemoattractants for neutrophils and macrophages [86,87]. Indeed, there was an increase in macrophage recruitment to xenografts and Matrigel plugs containing these pro-inflammatory fibroblasts [81]. A similar pro-inflammatory signature was observed in fibroblasts isolated from human cervical cancers, and antiestrogen treatment of these cervical CAFs revealed that the inflammation-associated gene expression is partially mediated by estrogen [88]. Our own unpublished results have identified a strong pro-inflammatory signature in the cervical stroma of estrogen-treated *K14E6/E7* mice [89]. It will be important to further define the role of the HPV-positive epithelia in inducing pro-inflammatory cytokines and chemokines, as well as their role in the stroma during HPV infection and carcinogenesis.

In addition to epithelial-derived IL-1B as described above [81], several other mechanisms have been proposed for induction of inflammation and immune cell recruitment by HPV-positive epithelial cells. For instance, it was reported that HPV-positive epithelial cells create a pro-tumorigenic and inflammatory microenvironment by secreting IL-6 [90,91]. The IL-6 secreted from HPV-positive epithelial cells induces expression of the chemokine C-C motif chemokine ligand 20 (CCL20) in stromal fibroblasts to attract pro-inflammatory T helper 17 (Th17) cells [92]. Xenograft studies comparing cervical cell lines concluded that the HPV-positive HeLa and SiHa cell xenografts recruited significantly more inflammatory cells, particularly macrophages, than the HPV-negative C33A cell xenografts in vivo, and this was due to increased IL-6 and IL-8 secretion from HPV-positive epithelial cells [93]. Tumor-associated macrophages in the stroma of HPV-positive tumors have been shown to secrete IL-10 in order to suppress antitumor T cell responses and create a pro-tumorigenic microenvironment [94,95].

Inflammatory cell recruitment by the HPV-positive epithelium was also observed in HPV transgenic mice. In *K14HPV16* mice, a significant influx of mast cells that secrete MMP-9 was observed in the microenvironment, which was necessary for promoting angiogenesis and epithelial proliferation in the skin [69,96]: Interestingly, the MMP-9 appeared to be mainly supplied by infiltrating immune cells, including mast cells, neutrophils, and macrophages, and not the epithelial cells [96]. While the individual contributions of the HPV viral proteins to inflammation and immune cell recruitment have not been fully elucidated, the HPV16 E7 protein alone in *K14E7* mice is sufficient for leukocyte trafficking to the skin and this function depends on its interaction with the pRb tumor suppressor [97,98]. An independent study found that the HPV16 E7 expressed in *K14E7* mouse skin is sufficient to recruit mast cells through elicitation of CCL cytokines, CCL2 and CCL5, and that this contributes to the oncogene driving establishment of an immunosuppressive environment within the skin, in a manner that is dependent upon the induction of epithelial hyperplasia by E7 [97]. Therefore, HPV-positive cells coordinate with the surrounding stroma to elicit a pro-inflammatory microenvironment, which in turn facilitates the immune and inflammatory infiltrate observed in the stroma of HPV-associated lesions and cancers [71]. Further elucidation of the role of HPV in promoting epithelial-to-stromal crosstalk in these processes will enhance our understanding of HPV-driven carcinogenesis.

### 3.2. Effects of the Stromal Microenvironment on HPV-Positive Epithelia

Upon HPV infection, it is clear that the stratified epithelium initiates communication with the underlying stroma. Presumably in response to this communication, the microenvironment reciprocates contact through various stromal-to-epithelial signaling events. While these processes are still not well understood, there is evidence that the stroma plays a role in HPV-positive epithelial cell growth and disease initiation and maintenance.

#### 3.2.1. Stromal Effects on Growth and Differentiation of HPV-Positive Epithelia

**Figure 3:**
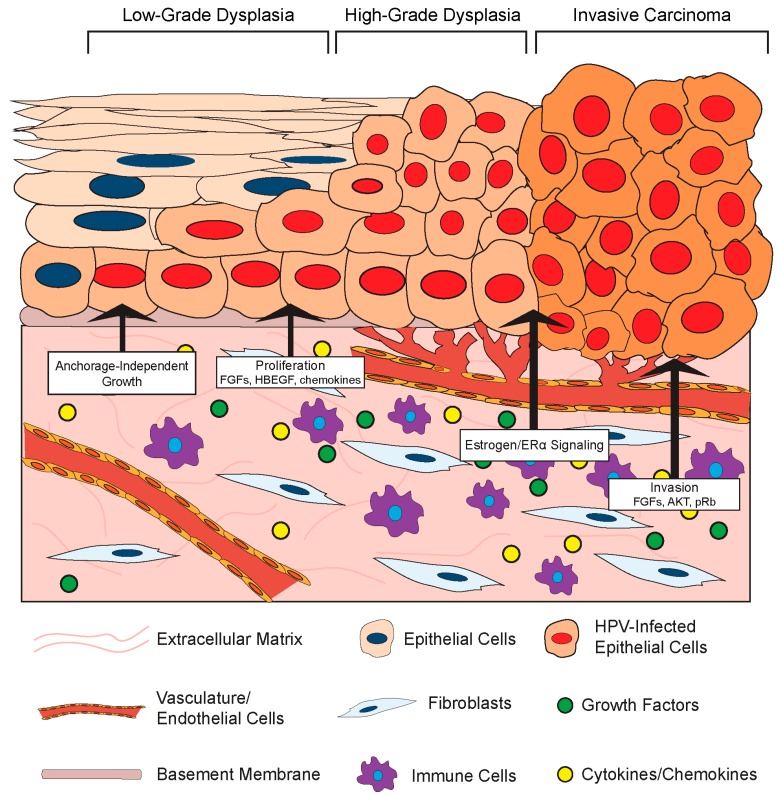
Effects of the stroma on HPV-positive epithelium. The HPV-infected epithelium undergoes neoplastic progression from low-grade to high-grade dysplasia and eventually invasive carcinoma. Key stromal-to-epithelial communication events between the stroma and the HPV-positive epithelium, as well as the reported paracrine factors involved in these processes, are depicted (large black arrows). The location of signaling events within the process of neoplastic progression is not necessarily reflective of their temporal activities. ERα: estrogen receptor α; HBEGF: heparin-binding epidermal growth factor-like growth factor; pRb: retinoblastoma protein.

Given its role in normal epithelial cell biology, it is not surprising that the stromal compartment also affects the growth and differentiation of HPV-infected epithelial cells (Figure 3). One such effect is HPV-induced cellular immortalization. High-risk HPVs can extend the life span of epithelial cells and ultimately induce cell immortalization via the functions of the E6 and E7 oncoproteins (Reviewed in [99]), and fibroblasts appear to cooperate in epithelial cell immortalization. For instance, feeder layers of growth-arrested murine 3T3 fibroblasts were required for epithelial cell immortalization by Rho kinase (ROCK) inhibitors [100,101]. Moreover, the presence of a feeder layer greatly enhanced the ability of the HPV E6/E7 proteins to immortalize cells in vitro [102]. Stromal cells also affect the growth properties of HPV-positive epithelial cells. Dermal fibroblasts induced anchorage-independent growth of HPV16-immortalized cervical epithelial cells, and this likely involved paracrine-acting factors since the effect did not require direct cell-to-cell contact [103]. In this same set of experiments, the presence of fibroblasts enhanced epithelial cell growth in organotypic rafts and increased expression of IL-1 in the co-cultured HPV16-positive epithelia. IL-1 is a cytokine implicated in epithelial–stromal interactions. In an illuminating body of work using organotypic rafts, Maas-Szabowski and colleagues defined a ‘double-paracrine’ epithelial–stromal signaling mechanism involving the IL-1 proteins, IL-1α (IL-1A) and IL-1B, that regulates epithelial differentiation and growth [43,49,104]. This work demonstrates that release of IL-1 by epithelial cells causes increased expression of keratinocyte growth factor (KGF; also known as FGF-7) in co-cultured fibroblasts, which subsequently feeds back in a paracrine manner to increase epithelial proliferation and tissue formation. Interestingly, IL-1A was reported to provide a selective growth advantage specifically to HPV16- and HPV18-positive cervical epithelial cells, and inhibited the growth of normal epithelial cells [105]. This paracrine signaling loop was further implicated in stromal regulation of growth and differentiation of HPV-positive epithelia in a series of studies by the McCance laboratory. In organotypic raft cultures composed of primary human foreskin keratinocytes immortalized with HPV16 E6 and E7 proteins and primary human foreskin fibroblasts, fibroblast-specific depletion of pRb increased KGF expression to facilitate epithelial invasion [106,107]. They also found that AKT kinase activation in the stromal fibroblasts increases the invasiveness of HPV-positive epithelia in a KGF-dependent manner [108]. These in vitro experiments indicate that both pRb and AKT in fibroblasts regulate the invasion of HPV-positive epithelia in a cell non-autonomous manner, and provide direct experimental evidence that the stroma regulates growth properties of the HPV-infected stratified epithelia.

Fibroblast growth factors are a family of secreted factors that are significant players in communication between the stroma and epithelium and are involved in processes ranging from development to cancer [109]. There is evidence that FGFs and their receptors may facilitate HPV-mediated carcinogenesis through a variety of mechanisms involving stromal-to-epithelial crosstalk [110]. As outlined above, FGF-7 (or KGF) has been implicated in stromal regulation of HPV-positive epithelial invasion. There is also evidence that another FGF, FGF-2 (or bFGF) correlates with the invasive potential of HPV-positive epithelial cells. The effects of primary human fibroblasts isolated from cervical normal or cancer tissue on the invasive properties of normal keratinocytes or HPV16-immortalized keratinocytes were analyzed using an in vitro Matrigel invasion assay [111]. In this study, conditioned media from CAFs stimulated invasivity of epithelial cells and this was associated with increased expression of FGF-2. Interestingly, the pro-invasion effect of the CAF-conditioned media and more specifically FGF-2 was only observed with HPV-positive epithelial cells. Conversely, conditioned media from normal fibroblasts inhibited penetration, and this inhibitory effect was mediated by transforming growth factor-β (TGF-β). From these studies, the authors concluded that the paracrine effects mediated by fibroblasts were due to an increased sensitivity of HPV-positive epithelial cells to FGF-2 and TGF-β, thus increasing their mobility and invasive phenotype. Other factors besides FGFs are also implicated. Human cervical fibroblasts also increased the invasiveness of the HPV18-postive cervical cancer cell line SKG-II by increasing MMP expression in in vitro Matrigel invasion assays [58]. Another group has also reported that CAFs induce migration of HPV-positive cervical cancer cells more than normal fibroblasts, and attributed this effect to differences in extracellular matrix remodeling [56]. Co-culture of HPV+ cervical ME180 and CaSki cancer epithelial cells with CAFs in vitro also enhanced epithelial cell proliferation, and this was dependent on production of an epidermal growth factor receptor (EGFR) ligand, the heparin-binding epidermal growth factor-like growth factor (HBEGF) [112]. Therefore, while a complete understanding of the specific interactions between the stroma and HPV-positive cells is still lacking, it is clear that the microenvironment and cells contained within can have a unique effect on the growth and proliferation of the epithelium when HPV or HPV oncogenes are present.

#### 3.2.2. Stromal Effects on Disease in HPV-Associated Cancer Models

Virus-associated carcinogenesis can be studied in vivo using xenografts as well as genetically engineered murine models [113]. As introduced in previous sections, there are two main HPV transgenic mouse models in which expression of HPV genes; primarily, the E6 and E7 oncogenes are directed specifically to epithelial cells by placing the viral genes under the transcriptional control of K14 transcriptional promoter. Initial studies of HPV transgenic mice revealed that expression of HPV16 E6 and E7 in the cervical epithelium is necessary but not sufficient to cause cervical cancer, which mirrors human data. Subsequent studies identified the female hormone estrogen (17β-estradiol) as a necessary co-factor for HPV-associated cervical carcinogenesis in both murine models [114,115]. In transgenic mice, treatment with 17-β-estradiol and expression of its receptor ERα are required for the onset, maintenance, and progression of neoplastic disease [114,115,116,117]. Furthermore, continuous treatment with estrogen signaling inhibitors promotes regression of cervical cancer and precancerous lesions, and prevents their recurrence, in HPV transgenic mice [118,119,120,121]. Human epidemiological data also correlate higher estrogen levels and an increased risk factor for cervical cancer [122,123]. The following section focuses on observations of stroma-to-epithelium interactions in vivo using both HPV-positive cervical cancer xenografts and the above referenced animal models of HPV-associated cancers. Emerging evidence linking the requirement for estrogen in cervical carcinogenesis to the stromal compartment will also be discussed.

Several experiments using xenografts have identified positive effects of fibroblasts on HPV-positive epithelial cells. In one set of experiments, xenografts of the HPV-positive cervical cancer cell line ME180 were significantly larger when the epithelial cells were co-inoculated with cervical cancer CAFs or mouse embryonic fibroblasts compared to epithelial cells alone [112]. The positive effect of fibroblasts on epithelial cell growth was mediated by the EGFR ligand, HB-EGF, expressed by the fibroblasts in response to PDGF secreted by HPV-positive epithelial cells [80]. Validating the HB-EGF-dependent mechanism, fibroblasts isolated from HB-EGF-null mice failed to positively affect ME180 xenograft growth. Notably, ME180 xenografts in nude mice rarely metastasize. However, lymph node metastases were observed in mice injected with xenografts composed of both ME180 cells and cervical cancer CAFs [124], suggesting that the fibroblasts provided a microenvironment favorable to HPV-positive epithelial cell migration and metastasis.

Another set of xenograft experiments analyzed the effect of stromal cells on the growth of PDSC5 cells, which are a murine HPV16-positive squamous cell carcinoma cell line derived from the skin of *K14HPV16* mice. In a study by Erez and colleagues, dermal fibroblasts were isolated from areas adjacent to normal skin in non-transgenic mice and dysplastic skin lesions spontaneously arising in *K14HPV16* transgenic mice, and their gene expression transcriptomes compared [81]. The CAFs isolated from the stroma adjacent to HPV-transgenic skin exhibited a significant pro-inflammatory signature composed of several pro-angiogenic cytokines and chemokines. Xenografts containing PDSC5 cells and CAFs or normal dermal fibroblasts were generated in mice. Similar to the results in ME180 cervical cancer cell xenografts, the presence of the pro-inflammatory CAFs increased the growth rate, size, and vascularization of PDSC5 skin cancer cell xenografts. In the PDSC5 xenograft model, expression of the cysteine protease cathepsin C by fibroblasts and leukocytes in the stromal compartment was necessary for tumor growth and angiogenesis [125]. Collectively, these results suggest that stromal cells can actively influence the in vivo growth of HPV-positive epithelial cells derived from various anatomical sites.

There is also evidence that the stroma influences de novo cervical cancers in transgenic mouse models of HPV-associated cervical cancer. In estrogen-treated *K14HPV16* mice, a paracrine loop between the epithelia and stroma involving PDGF and FGFs was identified [80]. In this study, both FGF-2 and FGF-7 were expressed by fibroblasts in response to PDGF secreted by the HPV-positive epithelia. When estrogen-treated *K14HPV16* mice were treated with imatinib to inhibit PDGF signaling, FGF-2 and FGF-7 expression was decreased in the stroma. Imatinib treatment reduced proliferation and increased apoptosis in the epithelium and effectively delayed progression and reduced in vivo cervical cancer growth. These effects were linked to a reduction in angiogenesis driven by fibroblast-derived FGFs. Expression of FGF-2 was similarly increased in the stroma of human cervical cancers. These experiments clearly define a role for the microenvironment in promoting HPV-associated cervical cancer growth through paracrine mechanisms.

In our own studies, we have made the important observation that estrogen contributes to cervical carcinogenesis by signaling through the underlying stroma. An extensive body of work by Cunha and colleagues had previously established that stromal–epithelial interactions mediate the effects of estrogen on morphogenesis of the female reproductive tract, and more specifically that stromal estrogen receptor α (ERα) facilitates the proliferative effects of estrogen on the epithelium [126,127,128,129]. In line with these observations, we found that neoplastic cervical disease in *K14E7* mice requires the continuous expression of ERα in the cervical stroma [130]. Stroma-specific deletion of ERα in the cervices of estrogen-treated HPV transgenic mice resulted in a hypoplastic epithelium with significantly reduced proliferation, despite the continued expression of ERα in the epithelial compartment. Moreover, the status of stromal ERα highly correlated with cervical disease, and ERα ablation in the stroma of estrogen-treated HPV transgenic mice significantly reduced cervical disease in the epithelia, and most mice had no signs of disease. These results revealed that the ability of estrogen to induce cervical disease in the HPV-positive epithelium involves estrogen signaling through the stromal compartment.

In a parallel study analyzing human cervical tissue samples spanning neoplastic disease progression from normal cervix to cervical cancers, ERα expression became progressively confined to the stromal compartment but was lost in epithelial cancer cells [131]. Fibroblasts were subsequently identified as being the predominant ERα-positive cell type in the stroma of human cervical cancers, a finding validated by other studies [88]. Similar to previous observations reporting a pro-inflammatory signature in fibroblasts adjacent to *K14HPV16* skin [81], human cervical cancer-associated fibroblasts likewise showed increased pro-tumorigenic and inflammation-associated gene expression [88]. Interestingly, the expression of several pro-tumorigenic genes in ex vivo cultured cervical CAFs was decreased upon treatment antiestrogen drugs, suggesting that stromal fibroblasts can indeed mediate estrogen-dependent signaling in the human cervix [88]. Collectively, these in vivo studies highlight that estrogen signaling in the cervical stroma drives cervical carcinogenesis in the HPV-positive cervical epithelium. We hypothesize that a unique repertoire of paracrine-acting factors is elicited by estrogen in the stroma of HPV transgenic mice that promote cervical cancer development in the adjacent epithelium, and current studies are underway to identify these factors. Nonetheless, there is increasing evidence that the stroma plays a pivotal role in the dynamics and progression of HPV-driven cervical cancer development.

## 4. Potential Mechanisms of Bidirectional Crosstalk

The functional consequences of HPV-induced epithelial–stromal crosstalk have been discussed throughout this review, and include influences on the HPV life cycle and virus-induced disease. Many of the factors implicated in the crosstalk are proteins secreted into the extracellular space (e.g., growth factors, cytokines/chemokines, proteases). In many cases, however, the mechanism for crosstalk has not been elucidated. One emerging mechanism to explain the communication between tissue compartments is the reciprocal secretion and uptake of extracellular vesicles by epithelial and stromal cells. Exosomes are one particular type of vesicle that has been extensively studied in the context of cancer biology [132]. Exosomes are 40–150 nm-sized vesicles composed of a lipid bilayer and containing intracellular cargo such as proteins, RNA, and/or DNA, all of which can be delivered to recipient cells. The exchange of genetic material, in the form of mRNA and miRNAs, is a particularly potent mechanism to modulate gene expression and function in recipient cells [133]. Importantly, viruses can modulate the vesicle profile of infected cells [54]. For instance, nasopharyngeal carcinoma cells harboring the oncogenic gammaherpesvirus Epstein–Barr virus (EBV) release exosomes that contain viral proteins and miRNAs, as well as signal transduction molecules, and were shown to activate pro-tumorigenic signaling pathways in recipient cells [134]. Several recent reports indicate that HPVs modulate exosome secretion and content. Exosomes secreted from keratinocytes expressing HPV E6 and E7 oncogenes contained several antiapoptotic proteins [135] and unique miRNA expression patterns [136,137,138]. While one report did not find evidence for E6 or E7 protein in exosomes [135], both E6 and E7 transcripts were detected in exosomes secreted from HPV-positive keratinocytes and cervical cancer cells in a more recent study [136]. The possibility that E6 and E7 transcripts and/or proteins may be transferred to cells via microvesicles would not only provide a potential mechanistic basis for epithelial–stromal crosstalk, but would also represent a major paradigm shift in our understanding of HPV-associated disease. In this regard, reports from one lab that E6 and E7 can be detected in the extracellular space and purified protein can be internalized by endothelial cells is particularly intriguing [139,140,141]. Furthermore, our own recent unpublished studies indicate that in vivo expression of the HPV oncogenes in the cervical epithelium has a profound, cell non-autonomous effect on global gene expression in nearby stromal cells [89]. It is not currently known whether or how microvesicles secreted from HPV-positive epithelial cells affect the function of surrounding cells, and such experiments will be crucial to dissecting their role in epithelial–stromal communication. A complete dissection of the mechanisms that underlie HPV-induced crosstalk between the stratified epithelium and the stroma will be important in fully understanding how HPV contributes to disease.

## 5. Conclusions and Future Perspectives

In this review, we have outlined the current knowledge of key interactions between human papillomaviruses and the underlying stroma. Established and emerging evidence indicate that bidirectional crosstalk between HPV-infected epithelial cells and the microenvironment contribute to the HPV life cycle (Figure 1), stromal architecture, angiogenesis, and inflammation (Figure 2), as well as growth of the epithelium and disease progression (Figure 3). While the role of the stroma in HPV infection and disease is increasingly appreciated, future studies are necessary to provide a complete picture of the reciprocal interactions between the two tissue compartments and the role of HPV in these processes. Organotypic cultures have historically been an important tool to study epithelial–stromal interactions and HPV infection in vitro [45,46,104] and will continue to provide a relevant experimental platform for future studies. Likewise, genetically engineered mouse models of HPV-associated disease [113] will allow interrogation of compartment-specific cell types and signaling pathways and their role in carcinogenesis in various anatomical sites. In addition to the animal model in which the rodent *Mastomys coucha* is naturally infected with *Mastomys natalensis* papillomavirus (MnPV) [142], the exciting discovery of a murine papillomavirus (MmuPV1) [143] will allow the ability to study the natural course of papillomavirus infections and disease progression in tractable and genetically modifiable hosts, a traditionally and historically elusive endeavor in the field. Our laboratory has already used the murine MmuPV1 model to study papillomavirus-associated skin disease [144], and future studies are underway to study cervical and oral disease induced by MmuPV1. The MmuPV1-based infection models, together with pre-existing HPV transgenic mouse models, will allow future studies necessary to understanding in vivo mechanisms of epithelial–stromal crosstalk during papillomavirus-induced disease.

## References

[1] De Martel C., Ferlay J., Franceschi S., Vignat J., Bray F., Forman D., Plummer M. (2012). Global burden of cancers attributable to infections in 2008: A review and synthetic analysis. Lancet Oncol..

[2] Mirvish E.D., Shuda M. (2016). Strategies for human tumor virus discoveries: From microscopic observation to digital transcriptome subtraction. Front. Microbiol..

[3] Zur Hausen H. (2009). Papillomaviruses in the causation of human cancers—A brief historical account. Virology.

[4] Bernard H.U., Burk R.D., Chen Z., van Doorslaer K., zur Hausen H., de Villiers E.M. (2010). Classification of papillomaviruses (PVs) based on 189 PV types and proposal of taxonomic amendments. Virology.

[5] Van Doorslaer K., Li Z., Xirasagar S., Maes P., Kaminsky D., Liou D., Sun Q., Kaur R., Huyen Y., McBride A.A. (2017). The papillomavirus episteme: A major update to the papillomavirus sequence database. Nucleic Acids Res..

[6] Satterwhite C.L., Torrone E., Meites E., Dunne E.F., Mahajan R., Ocfemia M.C., Su J., Xu F., Weinstock H. (2013). Sexually transmitted infections among us women and men: Prevalence and incidence estimates, 2008. Sex. Transm. Dis..

[7] Baseman J.G., Koutsky L.A. (2005). The epidemiology of human papillomavirus infections. J. Clin. Virol..

[8] Cogliano V., Baan R., Straif K., Grosse Y., Secretan B., El Ghissassi F., WHO International Agency for Research on Cancer (2005). Carcinogenicity of human papillomaviruses. Lancet Oncol..

[9] D’Souza G., Kreimer A.R., Viscidi R., Pawlita M., Fakhry C., Koch W.M., Westra W.H., Gillison M.L. (2007). Case-control study of human papillomavirus and oropharyngeal cancer. N. Engl. J. Med..

[10] Hoots B.E., Palefsky J.M., Pimenta J.M., Smith J.S. (2009). Human papillomavirus type distribution in anal cancer and anal intraepithelial lesions. Int. J. Cancer.

[11] Munoz N., Bosch F.X., Castellsague X., Diaz M., de Sanjose S., Hammouda D., Shah K.V., Meijer C.J. (2004). Against which human papillomavirus types shall we vaccinate and screen? The international perspective. Int. J. Cancer.

[12] Frazer I.H., Leggatt G.R., Mattarollo S.R. (2011). Prevention and treatment of papillomavirus-related cancers through immunization. Annu. Rev. Immunol..

[13] Jemal A., Simard E.P., Dorell C., Noone A.M., Markowitz L.E., Kohler B., Eheman C., Saraiya M., Bandi P., Saslow D. (2013). Annual report to the nation on the status of cancer, 1975–2009, featuring the burden and trends in human papillomavirus (HPV)-associated cancers and HPV vaccination coverage levels. J. Natl. Cancer Inst..

[14] Rodriguez A.C., Schiffman M., Herrero R., Hildesheim A., Bratti C., Sherman M.E., Solomon D., Guillen D., Alfaro M., Morales J. (2010). Longitudinal study of human papillomavirus persistence and cervical intraepithelial neoplasia grade 2/3: Critical role of duration of infection. J. Natl. Cancer Inst..

[15] Bodily J., Laimins L.A. (2011). Persistence of human papillomavirus infection: Keys to malignant progression. Trends Microbiol..

[16] Moscicki A.B., Schiffman M., Burchell A., Albero G., Giuliano A.R., Goodman M.T., Kjaer S.K., Palefsky J. (2012). Updating the natural history of human papillomavirus and anogenital cancers. Vaccine.

[17] Munoz N., Hernandez-Suarez G., Mendez F., Molano M., Posso H., Moreno V., Murillo R., Ronderos M., Meijer C., Munoz A. (2009). Persistence of HPV infection and risk of high-grade cervical intraepithelial neoplasia in a cohort of colombian women. Br. J. Cancer.

[18] Schiffman M., Rodriguez A.C., Chen Z., Wacholder S., Herrero R., Hildesheim A., Desalle R., Befano B., Yu K., Safaeian M. (2010). A population-based prospective study of carcinogenic human papillomavirus variant lineages, viral persistence, and cervical neoplasia. Cancer Res..

[19] Luft F., Klaes R., Nees M., Durst M., Heilmann V., Melsheimer P., von Knebel Doeberitz M. (2001). Detection of integrated papillomavirus sequences by ligation-mediated PCR (DIPS-PCR) and molecular characterization in cervical cancer cells. Int. J. Cancer.

[20] Wentzensen N., Ridder R., Klaes R., Vinokurova S., Schaefer U., Doeberitz M. (2002). Characterization of viral-cellular fusion transcripts in a large series of HPV16 and 18 positive anogenital lesions. Oncogene.

[21] Jang M.K., Shen K., McBride A.A. (2014). Papillomavirus genomes associate with BRD4 to replicate at fragile sites in the host genome. PLoS Pathog..

[22] Jeon S., Lambert P.F. (1995). Integration of human papillomavirus type 16 DNA into the human genome leads to increased stability of E6 and E7 mRNAs: Implications for cervical carcinogenesis. Proc. Natl. Acad. Sci. USA.

[23] Ziegert C., Wentzensen N., Vinokurova S., Kisseljov F., Einenkel J., Hoeckel M., von Knebel Doeberitz M. (2003). A comprehensive analysis of HPV integration loci in anogenital lesions combining transcript and genome-based amplification techniques. Oncogene.

[24] Jeon S., Allen-Hoffmann B.L., Lambert P.F. (1995). Integration of human papillomavirus type 16 into the human genome correlates with a selective growth advantage of cells. J. Virol..

[25] Munger K., Baldwin A., Edwards K.M., Hayakawa H., Nguyen C.L., Owens M., Grace M., Huh K. (2004). Mechanisms of human papillomavirus-induced oncogenesis. J. Virol..

[26] Zur Hausen H. (2000). Papillomaviruses causing cancer: Evasion from host-cell control in early events in carcinogenesis. J. Natl. Cancer Inst..

[27] Schiffman M., Castle P.E., Jeronimo J., Rodriguez A.C., Wacholder S. (2007). Human papillomavirus and cervical cancer. Lancet.

[28] Walboomers J.M., Jacobs M.V., Manos M.M., Bosch F.X., Kummer J.A., Shah K.V., Snijders P.J., Peto J., Meijer C.J., Muñoz N. (1999). Human papillomavirus is a necessary cause of invasive cervical cancer worldwide. J. Pathol..

[29] Bhowmick N.A., Neilson E.G., Moses H.L. (2004). Stromal fibroblasts in cancer initiation and progression. Nature.

[30] Quail D.F., Joyce J.A. (2013). Microenvironmental regulation of tumor progression and metastasis. Nat. Med..

[31] Gius D., Funk M.C., Chuang E.Y., Feng S., Huettner P.C., Nguyen L., Bradbury C.M., Mishra M., Gao S., Buttin B.M. (2007). Profiling microdissected epithelium and stroma to model genomic signatures for cervical carcinogenesis accommodating for covariates. Cancer Res..

[32] De Villiers E.M. (2013). Cross-roads in the classification of papillomaviruses. Virology.

[33] Zheng Z.M., Baker C.C. (2006). Papillomavirus genome structure, expression, and post-transcriptional regulation. Front. Biosci..

[34] Doorbar J., Egawa N., Griffin H., Kranjec C., Murakami I. (2015). Human papillomavirus molecular biology and disease association. Rev. Med. Virol..

[35] Pyeon D., Pearce S.M., Lank S.M., Ahlquist P., Lambert P.F. (2009). Establishment of human papillomavirus infection requires cell cycle progression. PLoS Pathog..

[36] Flores E.R., Allen-Hoffmann B.L., Lee D., Lambert P.F. (2000). The human papillomavirus type 16 E7 oncogene is required for the productive stage of the viral life cycle. J. Virol..

[37] Moody C.A., Laimins L.A. (2009). Human papillomaviruses activate the ATM DNA damage pathway for viral genome amplification upon differentiation. PLoS Pathog..

[38] Rheinwald J.G., Green H. (1975). Serial cultivation of strains of human epidermal keratinocytes: The formation of keratinizing colonies from single cells. Cell.

[39] Llames S., Garcia-Perez E., Meana A., Larcher F., del Rio M. (2015). Feeder layer cell actions and applications. Tissue Eng. Part B Rev..

[40] Dall K.L., Scarpini C.G., Roberts I., Winder D.M., Stanley M.A., Muralidhar B., Herdman M.T., Pett M.R., Coleman N. (2008). Characterization of naturally occurring HPV16 integration sites isolated from cervical keratinocytes under noncompetitive conditions. Cancer Res..

[41] Lee D., Lambert P. (2017). Maintenance of HPV genomes in NOKs cells requires fibroblast feeder layer. McArdle Laboratory for Cancer Research, University of Wisconsin-Madison, Madison, WI, USA.

[42] Werner S., Smola H. (2001). Paracrine regulation of keratinocyte proliferation and differentiation. Trends Cell Biol..

[43] Maas-Szabowski N., Shimotoyodome A., Fusenig N.E. (1999). Keratinocyte growth regulation in fibroblast cocultures via a double paracrine mechanism. J. Cell Sci..

[44] Longworth M.S., Laimins L.A. (2004). Pathogenesis of human papillomaviruses in differentiating epithelia. Microbiol. Mol. Biol. Rev..

[45] Meyers C., Laimins L.A. (1994). In vitro systems for the study and propagation of human papillomaviruses. Curr. Top. Microbiol. Immunol..

[46] Lambert P.F., Ozbun M.A., Collins A., Holmgren S., Lee D., Nakahara T. (2005). Using an immortalized cell line to study the HPV life cycle in organotypic “raft” cultures. Methods Mol. Med..

[47] Lee D., Norby K., Hayes M., Chiu Y.F., Sugden B., Lambert P.F. (2016). Using organotypic epithelial tissue culture to study the human papillomavirus life cycle. Curr. Protoc. Microbiol..

[48] El Ghalbzouri A., Ponec M. (2004). Diffusible factors released by fibroblasts support epidermal morphogenesis and deposition of basement membrane components. Wound Repair Regen..

[49] Maas-Szabowski N., Stark H.J., Fusenig N.E. (2000). Keratinocyte growth regulation in defined organotypic cultures through IL-1-induced keratinocyte growth factor expression in resting fibroblasts. J. Investig. Dermatol..

[50] Smola H., Stark H.J., Thiekotter G., Mirancea N., Krieg T., Fusenig N.E. (1998). Dynamics of basement membrane formation by keratinocyte-fibroblast interactions in organotypic skin culture. Exp. Cell Res..

[51] Smola H., Thiekotter G., Fusenig N.E. (1993). Mutual induction of growth factor gene expression by epidermal-dermal cell interaction. J. Cell Biol..

[52] Schumacher M., Schuster C., Rogon Z.M., Bauer T., Caushaj N., Baars S., Szabowski S., Bauer C., Schorpp-Kistner M., Hess J. (2014). Efficient keratinocyte differentiation strictly depends on JNK-induced soluble factors in fibroblasts. J. Investig. Dermatol..

[53] Werner S., Krieg T., Smola H. (2007). Keratinocyte-fibroblast interactions in wound healing. J. Investig. Dermatol..

[54] Schorey J.S., Harding C.V. (2016). Extracellular vesicles and infectious diseases: New complexity to an old story. J. Clin. Investig..

[55] Pickard A., McDade S.S., McFarland M., McCluggage W.G., Wheeler C.M., McCance D.J. (2015). HPV16 down-regulates the insulin-like growth factor binding protein 2 to promote epithelial invasion in organotypic cultures. PLoS Pathog..

[56] Fullar A., Dudas J., Olah L., Hollosi P., Papp Z., Sobel G., Karaszi K., Paku S., Baghy K., Kovalszky I. (2015). Remodeling of extracellular matrix by normal and tumor-associated fibroblasts promotes cervical cancer progression. BMC Cancer.

[57] Kessenbrock K., Plaks V., Werb Z. (2010). Matrix metalloproteinases: Regulators of the tumor microenvironment. Cell.

[58] Sato T., Sakai T., Noguchi Y., Takita M., Hirakawa S., Ito A. (2004). Tumor-stromal cell contact promotes invasion of human uterine cervical carcinoma cells by augmenting the expression and activation of stromal matrix metalloproteinases. Gynecol. Oncol..

[59] Smola-Hess S., Pahne J., Mauch C., Zigrino P., Smola H., Pfister H.J. (2005). Expression of membrane type 1 matrix metalloproteinase in papillomavirus-positive cells: Role of the human papillomavirus (HPV) 16 and HPV8 E7 gene products. J. Gen. Virol..

[60] Kaewprag J., Umnajvijit W., Ngamkham J., Ponglikitmongkol M. (2013). HPV16 oncoproteins promote cervical cancer invasiveness by upregulating specific matrix metalloproteinases. PLoS ONE.

[61] Zhu D., Ye M., Zhang W. (2015). E6/E7 oncoproteins of high risk HPV-16 upregulate MT1-MMP, MMP-2 and MMP-9 and promote the migration of cervical cancer cells. Int. J. Clin. Exp. Pathol..

[62] Arbeit J.M., Munger K., Howley P.M., Hanahan D. (1994). Progressive squamous epithelial neoplasia in K14-human papillomavirus type 16 transgenic mice. J. Virol..

[63] Herber R., Liem A., Pitot H., Lambert P.F. (1996). Squamous epithelial hyperplasia and carcinoma in mice transgenic for the human papillomavirus type 16 E7 oncogene. J. Virol..

[64] Song S., Pitot H.C., Lambert P.F. (1999). The human papillomavirus type 16 E6 gene alone is sufficient to induce carcinomas in transgenic animals. J. Virol..

[65] Lambert P.F., Pan H., Pitot H.C., Liem A., Jackson M., Griep A.E. (1993). Epidermal cancer associated with expression of human papillomavirus type 16 E6 and E7 oncogenes in the skin of transgenic mice. Proc. Natl. Acad. Sci. USA.

[66] Strati K., Pitot H.C., Lambert P.F. (2006). Identification of biomarkers that distinguish human papillomavirus (HPV)-positive versus HPV-negative head and neck cancers in a mouse model. Proc. Natl. Acad. Sci. USA.

[67] Stelzer M.K., Pitot H.C., Liem A., Schweizer J., Mahoney C., Lambert P.F. (2010). A mouse model for human anal cancer. Cancer Prev. Res..

[68] Coussens L.M., Hanahan D., Arbeit J.M. (1996). Genetic predisposition and parameters of malignant progression in K14-HPV16 transgenic mice. Am. J. Pathol..

[69] Coussens L.M., Raymond W.W., Bergers G., Laig-Webster M., Behrendtsen O., Werb Z., Caughey G.H., Hanahan D. (1999). Inflammatory mast cells up-regulate angiogenesis during squamous epithelial carcinogenesis. Genes Dev..

[70] Smith-McCune K., Zhu Y.H., Hanahan D., Arbeit J. (1997). Cross-species comparison of angiogenesis during the premalignant stages of squamous carcinogenesis in the human cervix and K14-HPV16 transgenic mice. Cancer Res..

[71] Mazibrada J., Ritta M., Mondini M., De Andrea M., Azzimonti B., Borgogna C., Ciotti M., Orlando A., Surico N., Chiusa L. (2008). Interaction between inflammation and angiogenesis during different stages of cervical carcinogenesis. Gynecol. Oncol..

[72] Chen W., Li F., Mead L., White H., Walker J., Ingram D.A., Roman A. (2007). Human papillomavirus causes an angiogenic switch in keratinocytes which is sufficient to alter endothelial cell behavior. Virology.

[73] Lopez-Ocejo O., Viloria-Petit A., Bequet-Romero M., Mukhopadhyay D., Rak J., Kerbel R.S. (2000). Oncogenes and tumor angiogenesis: The HPV-16 E6 oncoprotein activates the vascular endothelial growth factor (VEGF) gene promoter in a p53 independent manner. Oncogene.

[74] Toussaint-Smith E., Donner D.B., Roman A. (2004). Expression of human papillomavirus type 16 E6 and E7 oncoproteins in primary foreskin keratinocytes is sufficient to alter the expression of angiogenic factors. Oncogene.

[75] Walker J., Smiley L.C., Ingram D., Roman A. (2011). Expression of human papillomavirus type 16 E7 is sufficient to significantly increase expression of angiogenic factors but is not sufficient to induce endothelial cell migration. Virology.

[76] Pilch H., Schlenger K., Steiner E., Brockerhoff P., Knapstein P., Vaupel P. (2001). Hypoxia-stimulated expression of angiogenic growth factors in cervical cancer cells and cervical cancer-derived fibroblasts. Int. J. Gynecol. Cancer.

[77] Huang T.H., Chu T.Y. (2014). Repression of mir-126 and upregulation of adrenomedullin in the stromal endothelium by cancer-stromal cross talks confers angiogenesis of cervical cancer. Oncogene.

[78] Martinez I., Gardiner A.S., Board K.F., Monzon F.A., Edwards R.P., Khan S.A. (2008). Human papillomavirus type 16 reduces the expression of microRNA-218 in cervical carcinoma cells. Oncogene.

[79] Zhu N., Zhang D., Xie H., Zhou Z., Chen H., Hu T., Bai Y., Shen Y., Yuan W., Jing Q. (2011). Endothelial-specific intron-derived miR-126 is down-regulated in human breast cancer and targets both VEGFA and PIK3R2. Mol. Cell. Biochem..

[80] Pietras K., Pahler J., Bergers G., Hanahan D. (2008). Functions of paracrine pdgf signaling in the proangiogenic tumor stroma revealed by pharmacological targeting. PLoS Med..

[81] Erez N., Truitt M., Olson P., Arron S.T., Hanahan D. (2010). Cancer-associated fibroblasts are activated in incipient neoplasia to orchestrate tumor-promoting inflammation in an NF-κB-dependent manner. Cancer Cell.

[82] Kanodia S., Fahey L.M., Kast W.M. (2007). Mechanisms used by human papillomaviruses to escape the host immune response. Curr. Cancer Drug Targets.

[83] Stanley M.A. (2012). Epithelial cell responses to infection with human papillomavirus. Clin. Microbiol. Rev..

[84] Mangino G., Chiantore M.V., Iuliano M., Fiorucci G., Romeo G. (2016). Inflammatory microenvironment and human papillomavirus-induced carcinogenesis. Cytokine Growth Factor Rev..

[85] Woodby B., Scott M., Bodily J. (2016). The interaction between human papillomaviruses and the stromal microenvironment. Prog. Mol. Biol. Transl. Sci..

[86] Chow M.T., Luster A.D. (2014). Chemokines in cancer. Cancer Immunol. Res..

[87] Keeley E.C., Mehrad B., Strieter R.M. (2010). CXC chemokines in cancer angiogenesis and metastases. Adv. Cancer Res..

[88] Kumar M.M., Davuluri S., Poojar S., Mukherjee G., Bajpai A.K., Bafna U.D., Devi U.K., Kallur P.P., Kshitish A.K., Jayshree R.S. (2016). Role of estrogen receptor α in human cervical cancer-associated fibroblasts: A transcriptomic study. Tumour Biol..

[89] Spurgeon M.E., Horswill M., den Boon J.A., Barthakur S., Forouzan O., Beebe D.J., Roopra A., Ahlquist P., Lambert P. (2017). Human papillomavirus oncogenes reprogram the cervical cancer microenvironment independently of and synergistically with estrogen.

[90] Pahne-Zeppenfeld J., Schroer N., Walch-Ruckheim B., Oldak M., Gorter A., Hegde S., Smola S. (2014). Cervical cancer cell-derived interleukin-6 impairs CCR7-dependent migration of MMP-9-expressing dendritic cells. Int. J. Cancer.

[91] Schroer N., Pahne J., Walch B., Wickenhauser C., Smola S. (2011). Molecular pathobiology of human cervical high-grade lesions: Paracrine STAT3 activation in tumor-instructed myeloid cells drives local MMP-9 expression. Cancer Res..

[92] Walch-Ruckheim B., Mavrova R., Henning M., Vicinus B., Kim Y.J., Bohle R.M., Juhasz-Boss I., Solomayer E.F., Smola S. (2015). Stromal fibroblasts induce CCL20 through IL6/C/EBPβ to support the recruitment of Th17 cells during cervical cancer progression. Cancer Res..

[93] Stone S.C., Rossetti R.A., Lima A.M., Lepique A.P. (2014). HPV associated tumor cells control tumor microenvironment and leukocytosis in experimental models. Immun. Inflamm. Dis..

[94] Lepique A.P., Daghastanli K.R., Cuccovia I.M., Villa L.L. (2009). HPV16 tumor associated macrophages suppress antitumor T cell responses. Clin. Cancer Res..

[95] Bolpetti A., Silva J.S., Villa L.L., Lepique A.P. (2010). Interleukin-10 production by tumor infiltrating macrophages plays a role in human papillomavirus 16 tumor growth. BMC Immunol..

[96] Coussens L.M., Tinkle C.L., Hanahan D., Werb Z. (2000). MMP-9 supplied by bone marrow-derived cells contributes to skin carcinogenesis. Cell.

[97] Bergot A.S., Ford N., Leggatt G.R., Wells J.W., Frazer I.H., Grimbaldeston M.A. (2014). HPV16-E7 expression in squamous epithelium creates a local immune suppressive environment via CCL2- and CCL5-mediated recruitment of mast cells. PLoS Pathog..

[98] Choyce A., Yong M., Narayan S., Mattarollo S.R., Liem A., Lambert P.F., Frazer I.H., Leggatt G.R. (2013). Expression of a single, viral oncoprotein in skin epithelium is sufficient to recruit lymphocytes. PLoS ONE.

[99] Munger K., Howley P.M. (2002). Human papillomavirus immortalization and transformation functions. Virus Res..

[100] Chapman S., McDermott D.H., Shen K., Jang M.K., McBride A.A. (2014). The effect of Rho kinase inhibition on long-term keratinocyte proliferation is rapid and conditional. Stem Cell Res. Ther..

[101] Liu X., Ory V., Chapman S., Yuan H., Albanese C., Kallakury B., Timofeeva O.A., Nealon C., Dakic A., Simic V. (2012). ROCK inhibitor and feeder cells induce the conditional reprogramming of epithelial cells. Am. J. Pathol..

[102] Fu B., Quintero J., Baker C.C. (2003). Keratinocyte growth conditions modulate telomerase expression, senescence, and immortalization by human papillomavirus type 16 E6 and E7 oncogenes. Cancer Res..

[103] Zheng J., Vaheri A. (1995). Human skin fibroblasts induce anchorage-independent growth of HPV-16-DNA-immortalized cervical epithelial cells. Int. J. Cancer.

[104] Maas-Szabowski N., Szabowski A., Stark H.J., Andrecht S., Kolbus A., Schorpp-Kistner M., Angel P., Fusenig N.E. (2001). Organotypic cocultures with genetically modified mouse fibroblasts as a tool to dissect molecular mechanisms regulating keratinocyte growth and differentiation. J. Investig. Dermatol..

[105] Woodworth C.D., McMullin E., Iglesias M., Plowman G.D. (1995). Interleukin 1α and tumor necrosis factor α stimulate autocrine amphiregulin expression and proliferation of human papillomavirus-immortalized and carcinoma-derived cervical epithelial cells. Proc. Natl. Acad. Sci. USA.

[106] Pickard A., Cichon A.C., Barry A., Kieran D., Patel D., Hamilton P., Salto-Tellez M., James J., McCance D.J. (2012). Inactivation of Rb in stromal fibroblasts promotes epithelial cell invasion. EMBO J..

[107] Pickard A., Cichon A.C., Menges C., Patel D., McCance D.J. (2012). Regulation of epithelial differentiation and proliferation by the stroma: A role for the retinoblastoma protein. J. Investig. Dermatol..

[108] Cichon A.C., Pickard A., McDade S.S., Sharpe D.J., Moran M., James J.A., McCance D.J. (2013). AKT in stromal fibroblasts controls invasion of epithelial cells. Oncotarget.

[109] Ornitz D.M., Itoh N. (2015). The fibroblast growth factor signaling pathway. Wiley Interdiscip. Rev. Dev. Biol..

[110] Arbeit J.M., Olson D.C., Hanahan D. (1996). Upregulation of fibroblast growth factors and their receptors during multi-stage epidermal carcinogenesis in K14-HPV16 transgenic mice. Oncogene.

[111] Turner M.A., Darragh T., Palefsky J.M. (1997). Epithelial-stromal interactions modulating penetration of matrigel membranes by HPV 16-immortalized keratinocytes. J. Investig. Dermatol..

[112] Murata T., Mizushima H., Chinen I., Moribe H., Yagi S., Hoffman R.M., Kimura T., Yoshino K., Ueda Y., Enomoto T. (2011). HB-EGF and PDGF mediate reciprocal interactions of carcinoma cells with cancer-associated fibroblasts to support progression of uterine cervical cancers. Cancer Res..

[113] Lambert P.F. (2016). Transgenic mouse models of tumor virus action. Annu. Rev. Virol..

[114] Arbeit J.M., Howley P.M., Hanahan D. (1996). Chronic estrogen-induced cervical and vaginal squamous carcinogenesis in human papillomavirus type 16 transgenic mice. Proc. Natl. Acad. Sci. USA.

[115] Riley R.R., Duensing S., Brake T., Munger K., Lambert P.F., Arbeit J.M. (2003). Dissection of human papillomavirus E6 and E7 function in transgenic mouse models of cervical carcinogenesis. Cancer Res..

[116] Brake T., Lambert P.F. (2005). Estrogen contributes to the onset, persistence, and malignant progression of cervical cancer in a human papillomavirus-transgenic mouse model. Proc. Natl. Acad. Sci. USA.

[117] Chung S.H., Wiedmeyer K., Shai A., Korach K.S., Lambert P.F. (2008). Requirement for estrogen receptor alpha in a mouse model for human papillomavirus-associated cervical cancer. Cancer Res..

[118] Chung S.H., Lambert P.F. (2009). Prevention and treatment of cervical cancer in mice using estrogen receptor antagonists. Proc. Natl. Acad. Sci. USA.

[119] Mehta F.F., Baik S., Chung S.H. (2017). Recurrence of cervical cancer and its resistance to progestin therapy in a mouse model. Oncotarget.

[120] Spurgeon M.E., Chung S.H., Lambert P.F. (2014). Recurrence of cervical cancer in mice after selective estrogen receptor modulator therapy. Am. J. Pathol..

[121] Yoo Y.A., Son J., Mehta F.F., DeMayo F.J., Lydon J.P., Chung S.H. (2013). Progesterone signaling inhibits cervical carcinogenesis in mice. Am. J. Pathol..

[122] Bronowicka-Klys D.E., Lianeri M., Jagodzinski P.P. (2016). The role and impact of estrogens and xenoestrogen on the development of cervical cancer. Biomed. Pharmacother..

[123] Chung S.H., Franceschi S., Lambert P.F. (2010). Estrogen and ERα: Culprits in cervical cancer?. Trends Endocrinol. Metab..

[124] Murata T., Mekada E., Hoffman R.M. (2017). Reconstitution of a metastatic-resistant tumor microenvironment with cancer-associated fibroblasts enables metastasis. Cell Cycle.

[125] Ruffell B., Affara N.I., Cottone L., Junankar S., Johansson M., DeNardo D.G., Korets L., Reinheckel T., Sloane B.F., Bogyo M. (2013). Cathepsin C is a tissue-specific regulator of squamous carcinogenesis. Genes Dev..

[126] Cooke P.S., Buchanan D.L., Young P., Setiawan T., Brody J., Korach K.S., Taylor J., Lubahn D.B., Cunha G.R. (1997). Stromal estrogen receptors mediate mitogenic effects of estradiol on uterine epithelium. Proc. Natl. Acad. Sci. USA.

[127] Cunha G.R. (1976). Stromal induction and specification of morphogenesis and cytodifferentiation of the epithelia of the mullerian ducts and urogenital sinus during development of the uterus and vagina in mice. J. Exp. Zool..

[128] Cunha G.R., Cooke P.S., Kurita T. (2004). Role of stromal-epithelial interactions in hormonal responses. Arch. Histol. Cytol..

[129] Kurita T., Cooke P.S., Cunha G.R. (2001). Epithelial-stromal tissue interaction in paramesonephric (Mullerian) epithelial differentiation. Dev. Biol..

[130] Chung S.H., Shin M.K., Korach K.S., Lambert P.F. (2013). Requirement for stromal estrogen receptor α in cervical neoplasia. Horm. Cancer.

[131] den Boon J.A., Pyeon D., Wang S.S., Horswill M., Schiffman M., Sherman M., Zuna R.E., Wang Z., Hewitt S.M., Pearson R. (2015). Molecular transitions from papillomavirus infection to cervical precancer and cancer: Role of stromal estrogen receptor signaling. Proc. Natl. Acad. Sci. USA.

[132] Kalluri R. (2016). The biology and function of exosomes in cancer. J. Clin. Investig..

[133] Valadi H., Ekstrom K., Bossios A., Sjostrand M., Lee J.J., Lotvall J.O. (2007). Exosome-mediated transfer of mRNAs and microRNAs is a novel mechanism of genetic exchange between cells. Nat. Cell Biol..

[134] Meckes D.G., Shair K.H., Marquitz A.R., Kung C.P., Edwards R.H., Raab-Traub N. (2010). Human tumor virus utilizes exosomes for intercellular communication. Proc. Natl. Acad. Sci. USA.

[135] Honegger A., Leitz J., Bulkescher J., Hoppe-Seyler K., Hoppe-Seyler F. (2013). Silencing of human papillomavirus (HPV) E6/E7 oncogene expression affects both the contents and the amounts of extracellular microvesicles released from HPV-positive cancer cells. Int. J. Cancer.

[136] Chiantore M.V., Mangino G., Iuliano M., Zangrillo M.S., De Lillis I., Vaccari G., Accardi R., Tommasino M., Columba Cabezas S., Federico M. (2016). Human papillomavirus E6 and E7 oncoproteins affect the expression of cancer-related microRNAs: Additional evidence in HPV-induced tumorigenesis. J. Cancer Res. Clin. Oncol..

[137] Harden M.E., Munger K. (2017). Human papillomavirus 16 E6 and E7 oncoprotein expression alters microRNA expression in extracellular vesicles. Virology.

[138] Honegger A., Schilling D., Bastian S., Sponagel J., Kuryshev V., Sultmann H., Scheffner M., Hoppe-Seyler K., Hoppe-Seyler F. (2015). Dependence of intracellular and exosomal micrornas on viral *E6*/*E7* oncogene expression in HPV-positive tumor cells. PLoS Pathog..

[139] Le Buanec H., D’Anna R., Lachgar A., Zagury J.F., Bernard J., Ittele D., d’Alessio P., Hallez S., Giannouli C., Burny A. (1999). HPV-16 E7 but not E6 oncogenic protein triggers both cellular immunosuppression and angiogenic processes. Biomed. Pharmacother..

[140] Le Buanec H., Lachgar A., D’Anna R., Zagury J.F., Bizzini B., Bernard J., Ittele D., Hallez S., Giannouli C., Burny A. (1999). Induction of cellular immunosuppression by the human papillomavirus type 16 E7 oncogenic protein. Biomed. Pharmacother..

[141] D’Anna R., Le Buanec H., Alessandri G., Caruso A., Burny A., Gallo R., Zagury J.F., Zagury D., D’Alessio P. (2001). Selective activation of cervical microvascular endothelial cells by human papillomavirus 16-E7 oncoprotein. J. Natl. Cancer Inst..

[142] Vinzon S.E., Braspenning-Wesch I., Muller M., Geissler E.K., Nindl I., Grone H.J., Schafer K., Rosl F. (2014). Protective vaccination against papillomavirus-induced skin tumors under immunocompetent and immunosuppressive conditions: A preclinical study using a natural outbred animal model. PLoS Pathog..

[143] Ingle A., Ghim S., Joh J., Chepkoech I., Bennett Jenson A., Sundberg J.P. (2011). Novel laboratory mouse papillomavirus (MusPV) infection. Vet. Pathol..

[144] Uberoi A., Yoshida S., Frazer I.H., Pitot H.C., Lambert P.F. (2016). Role of ultraviolet radiation in papillomavirus-induced disease. PLoS Pathog..

